# TLR2 Expression on Leukemic B Cells from Patients with Chronic Lymphocytic Leukemia

**DOI:** 10.1007/s00005-018-0523-9

**Published:** 2018-09-08

**Authors:** Agata Szymańska, Agnieszka Bojarska-Junak, Arkadiusz Drobiecki, Waldemar Tomczak, Jacek Roliński, Marek Hus, Iwona Hus

**Affiliations:** 1 Independent Clinical Transplantology Unit, Medical University of Lublin, Lublin, Poland; 2 Department of Clinical Immunology, Medical University of Lublin, Lublin, Poland; 3 Department of Hematology and Bone Marrow Transplantation, Holy Cross Cancer Center, Kielce, Poland; 4 Department of Hematooncology and Bone Marrow Transplantation, Medical University of Lublin, Lublin, Poland

**Keywords:** Chronic lymphocytic leukemia, Toll-like receptors, TLR2 expression, Prognostic factors

## Abstract

Antigenic stimulation is considered as a possible trigger of neoplastic transformation in chronic lymphocytic leukemia (CLL). B-cell receptor plays a key role in the interactions between the microenvironment and leukemic cells; however, an important role has also been attributed to Toll-like receptors (TLRs). It is believed that disorders of TLR expression may play a part in the pathogenesis of CLL. In this study, we investigated the potential role of TLR2 in CLL by analyzing its expression on leukemic B cells in correlation with clinical and laboratory parameters characterizing disease activity and patients’ immune status. We assessed the frequencies of TLR2^+^/CD19^+^ cells by the flow cytometry method in peripheral blood of 119 patients with CLL. The percentage of TLR2^+^/CD19^+^ cells was significantly lower in patients with CLL as compared to the healthy volunteers. There was also a lower percentage of TLR2^+^/CD19^+^ cells in CLL patients with poor prognostic factors, such as ZAP70 and/or CD38 expression, 17p and/or 11q deletion. On the other hand, among patients with del(13q14) associated with favorable prognosis, the percentage of TLR2^+^/CD19^+^ cells was higher than among those with del(11q22) and/or del(17p13) as well as in the control group. We found an association between low percentage of CD19^+^/CD5^+^/TLR2^+^ cells and shorter time to treatment. We also demonstrated the relationship between low percentage of CD19^+^/CD5^+^ TLR2-positive and overall survival (OS) of CLL patients. CLL patients with a proportion of 1.6% TLR2-positive B CD5^+^ cells (according to the receiver operating characteristic curve analysis) or more had a longer time to treatment and longer OS than the group with a lower percentage of TLR2 positive cells. To sum up, the results of the study suggest that low TLR2 expression is associated with poor prognosis in CLL patients. The monitoring of CD19^+^/CD5^+^/TLR2^+^ cells number may provide useful information on disease activity. Level of TLR2 expression on leukemic B cells may be an important factor of immunological dysfunction for patients with CLL. Our study suggests that TLR2 could becomes potential biological markers for the clinical outcome in patients with CLL.

## Introduction

Chronic lymphocytic leukemia (CLL) is characterized by the proliferation and accumulation of clonal B cells in bone marrow, peripheral blood, lymph nodes, spleen and more rarely extralymphatic organs. Most patients with CLL suffer from immune disorders, especially those associated with the impaired immune response (Ghia et al. 2008). According to current opinions, immune disorders may play a role in the pathogenesis of CLL, although the mechanism of the CLL development remains unexplained. It is hypothesized that factors derived from the external environment can induce proliferation of leukemic cells via numerous receptors, primarily B-cell receptor (BCR), but also chemokine receptors or cell adhesion molecules (Garcia-Muñoz et al. 2012; Ghia et al. 2008). Interactions between leukemic cells and microenvironment are thought to inhibit apoptosis, leading “in vivo” to prolongation of leukemic cells survival time and their accumulation in bone marrow, peripheral blood and lymphatic organs (Kostareli et al. 2012; Muzio et al. 2008). A process of rapid spontaneous apoptosis of leukemic cells observed in “in vitro” conditions confirms the significance of microenvironment for their survival. Stereotyped BCR sequences that were detected in leukemic cells might indicate the existence of a common antigen responsible for chaotic proliferation of leukemic cells (Murray et al. 2008). In addition to the key role of BCR in induction of leukemogenesis, an important function is also assigned to receptors recognizing pathogens, like Toll-like receptors (TLRs). It is believed that the disorders of TLR expression may contribute to the development of CLL (Muzio et al. 2009). TLRs are transmembrane proteins present on the surface and in endosomes of many cell types. Their stimulation influences the induction of the innate immune system, with particular importance of TLRs expressed on antigen-presenting cells (APC; dendritic cells, mast cells, macrophages and B cells) (Dajon et al. 2017; Meyer-Bahlburg and Rawlings 2008). TLR recognize the pathogen-associated molecular patterns (PAMP) (Grandjenette et al. 2007) and their ligands include lipopolysaccharides (LPS), DNA (mainly unmethylated CpG sequences), single and double-stranded RNA, peptidoglycan, zymosan or lipoteichoic acid derived from microorganisms such as bacteria, viruses, fungi or protozoa (Akira 2003). Depending on the ligand being recognized and the type of induced response, TLRs are characterized by a different localization in the cell as well as a different pool of effector and adaptor proteins, such as: MyD88, TRIF, TRAM (O’Neill and Bowie 2007). The final effect of the signal transduction is the activation of nuclear factor-κB transcription factor-dependent genes or type I interferon and pro-inflammatory cytokines (IL-1β, -2, -6, -8, -12, -15, -18), and tumor necrosis factor genes (Kawai and Akira 2011). As a consequence of TLR activation, there is also an increase in expression of adhesion and co-stimulatory molecules (CD40, CD80, CD86) (Chiron et al. 2008). Tissue macrophages after being activated by TLRs exhibit increased phagocytic activity as well as production of nitric oxide and reactive oxygen species. It has been found that macrophages lacking TLR2 and TLR4 or MyD88 protein expression have an impaired ability of phagocytosis of Gram-negative and Gram-positive bacteria (Blander and Medzhitov 2004). In addition to activating the innate immune system, TLR stimulation may also activate the adaptive immune system to combat pathogens. By stimulating APC, TLRs are involved in directing the adaptive immune system to T helper (Th)1 or Th2 response. Pathogens have developed the ability to activate TLR2 signal transduction as a defense mechanism by silencing Th1 responses and at the same time mobilizing Th2 responses (Damo et al. 2004). Stimulation of TLR2 on CD4^+^CD25^+^ regulatory T (Treg) cells by PAMP induces increased IL-10 release, leading to suppression of both humoral and cell-mediated immunity. This results in decreasing of Treg suppressor properties (Guangwei and Yong 2007; Netea et al. 2004).

Despite numerous studies on the factors responsible for malignant transformation and growth of leukemia cells, pathogenesis of CLL remains unknown. According to one of the hypothesis, bacterial and viral infections may constitute an etiological factor triggering the process of carcinogenesis in CLL justifies the studies on pathogen recognizing receptors such as TLRs. The role of TLRs expression in CLL pathogenesis remains undefined and requires critical analysis. Thus, it would be relevant to show whether TLRs in combination with established prognostic factors could improve risk stratification of CLL patients. In the current study, we examined the potential role of TLR2 in CLL by analyzing the level of TLR2 expression on CD19^+^/CD5^+^ cells in peripheral blood in correlation with clinical and laboratory parameters characterizing disease activity and patients’ immune status.

## Patients and Methods

### Patients

The study group included 119 patients (60 men and 59 women) aged 49–87 years (median 65) diagnosed with CLL at the Department of Hematooncology and Bone Marrow Transplantation of Medical University of Lublin and Department of Hematology and Bone Marrow Transplantation Holy Cross Cancer Center in Kielce.

The clinical stage of CLL was assessed based on the Rai staging system (Rai et al. 1975). Stage 0 was found in 25 patients, stage I in 37 patients, stage II in 36 patients, stage III in 13 patients and stage IV in 8 patients. The patients were divided into three groups: low risk (stage 0 according to Rai), intermediate-risk (stage I/II according to Rai) and high-risk (stage III/IV according to Rai). The control group consisted of 24 healthy donors, including 10 women and 14 men aged between 37 and 83 years (median: 57 years). Patient characteristics at the time of CLL diagnosis are summarized in Table 1. The study was approved by the Bioethics Committee of the Medical University of Lublin (decision No. KE 0254-150/2013). All patients gave written informed consent to participate in the study.

**Table 1:** Clinical characteristics of patients with CLL

	No. patients (%)
Rai stage	
Low risk (stage 0)	25 (21%)
Intermediate-risk (stage I/II)	73 (61%)
High-risk (stage III/IV)	21 (18%)
ZAP-70 (cut-off 20%)	
Positive (%)	51 (43%)
Negative (%)	68 (57%)
CD38 (cut-off 30%)	
Positive (%)	48 (40%)
Negative (%)	71 (60%)
Cytogenetic abnormalities	
del(17p13.1) and/or del(11q22.3)	49 (41.18%)
Isolated del(13q14)	35 (29.41%)
Without del(17p13.1) and del(11q22.3) and del(13q14)	29 (24.37%)
Not evaluated	6 (5.04%)
Patients requiring therapy	51 (42.9%)
Untreated patients	68 (57.1%)

	Median (min–max)
Age at diagnosis (years)	65 (49–87)
WBC count (G/L)	29.53 (8.72–330.56)
Lymphocyte count (G/L)	20.07 (5.51–317.85)
β_2_M (mg/dL)	2.39 (1.36–5.86)
LDH (IU/L)	355 (265–886)
Hemoglobin (g/dL)	13.6 (9.2–16.5)
Platelets (G/L)	174 (49–388)
CD19^+^/CD5^+^/ZAP-70^+^ cells (%)	24.64 (0.21–64.29)
CD19^+^/CD5^+^/CD38^+^ cells (%)	29.42 (0.22–80.90)

*WBC* white blood cell, *LDH* lactate dehydrogenase, *β2M* β2-microglobulin

### Cell Preparation

Peripheral blood samples were collected into EDTA tubes after diagnosis of CLL prior to initiation of treatment during routine diagnostic tests. Fresh peripheral blood samples were stained within 1–2 h and analyzed directly upon completion of staining process. Peripheral blood mononuclear cells were separated by density gradient centrifugation on Biocoll Separating Solution (Biochrom) for 25 min at 400×*g* at room temperature. Interphase cells were removed, washed twice, and resuspended in phosphate-buffered saline (PBS).

### Evaluation of the Percentage of TLR2^+^/CD19^+^ Cells

**Fig. 1:**
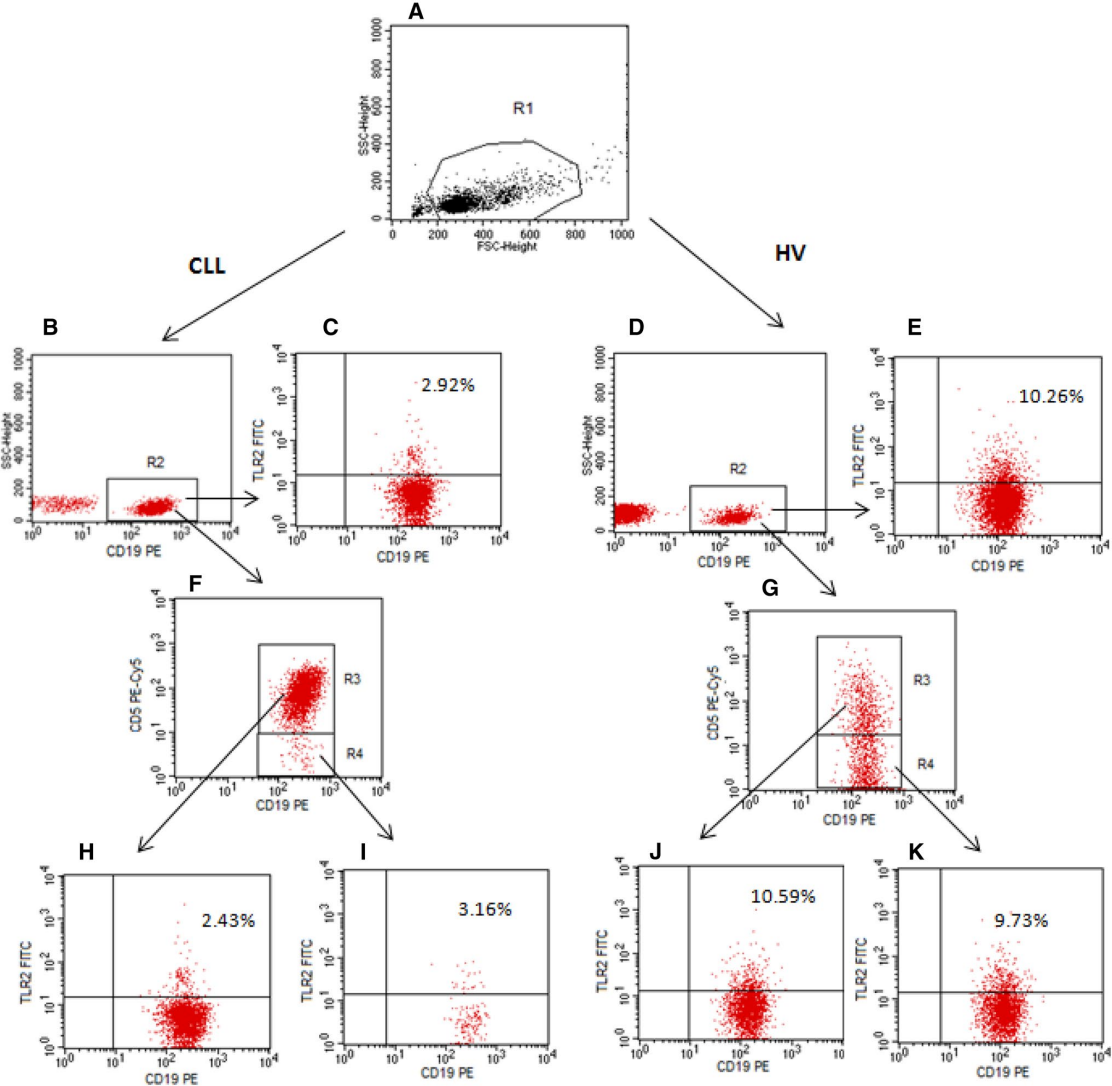
Representative dot plots of CLL patient and healthy volunteer (HV) illustrating the flow cytometry analysis method for the identification of B cells with TLR2 expression. **a** An acquisition gate was established based on FSC and SSC that included mononuclear cells (R1 region). **b, d** The R1 gated events were analyzed for CD19 PE staining, and the positive cells (CD19^+^) were gated (region R2). The dot plots **c, e** (CD19 PE vs. TLR2 FITC) were established by the combined gating of events using R1 and R2 regions. The number in the upper right quadrant on the dot plots **c, e** represents the percentage of CD19^+^/TLR2^+^ cells. Additional analysis for identification of CD19^+^/CD5^+^ cells with TLR2 expression was performed. **f, g** The R2 gated events (CD19^+^) were analyzed for CD5 PE-Cy5 staining, and the positive cells (CD19^+^/CD5^+^) were gated (region R3). Additionally, CD19^+^/CD5^−^ cells were gated (region R4). The dot plots **h, j** were established by the combined gating of events using R1, R2 and R3 regions. The dot plots **i, k** were established by the combined gating of events using R1, R2 and R4 regions. The number in the upper right quadrant on the dot plots **h, j** represents the percentage of CD19^+^/CD5^+^/TLR2^+^ cells. The dot plots **i, k** indicate CD19^+^/CD5^−^ cells positive for TLR2

The expression of surface antigens on B cells was evaluated using flow cytometry in accordance with the manufacturer’s recommended procedure. Mononuclear cells (1 × 10^6^) were labeled with anti-CD19 PE, anti-CD5 PE-Cy5 and anti-TLR2 (CD282) FITC monoclonal antibodies (BD Pharmingen, USA). After 20 min of incubation in the dark at room temperature unbound antibodies were washed twice with PBS solution, spinning cells for 5 min at 700×*g*. Cell suspension was analyzed by a flow cytometry. Evaluation of CD19^+^/CD5^+^/TLR2^+^ B cells was performed in BD FACSCalibur flow cytometer (BD Biosciences, USA). CellQuest Pro software was used for analysis and graphical presentation of data. For each analysis 20,000 events were acquired and analyzed. B CD5^+^ lymphocytes with TLR2 expression were analyzed within gated CD19^+^/CD5^+^ cells. Dot plots illustrating the analysis method for the identification of B CD5^+^ cells with membrane expression of TLR2 are presented in Fig. 1. In the experiment, the percentage of TLR2-positive CD19^+^CD5^+^ cells and the level of TLR2 expression on cells, indicated by the mean fluorescence intensity (MFI), were analyzed. The number of receptors per cell is directly associated to the intensity of the fluorescence, measured by flow cytometry (CellQuest Pro software) after incubation of the cells with antibodies. The expression of TLR2 was determined relative to the isotype control.

### Evaluation of ZAP-70 and CD38 Expression

Evaluation of ZAP-70 expression in CD19^+^/CD5^+^ leukemic cells in all tested samples was performed according to the previously described procedure (Hus et al. 2006) using monoclonal antibodies: anti-CD19 FITC, anti-ZAP-70 PE (clone 1E7.2) and anti-CD5 PE-Cy5 (BD Pharmingen, USA). Expression of CD38 on leukemic cells was evaluated using monoclonal antibodies anti-CD19 FITC, anti-CD38 PE and anti-CD5 PE-Cy5 (BD Pharmingen, USA). The cut-off point for leukemia cells with ZAP-70 expression was ≥ 20%, while for leukemia cells with CD38 expression it was ≥ 30%.

### Determination of CD19^+^ Lymphocytes Apoptosis by MitoTracker Red CMXRos (Chloromethyl-X-Rosamine)

In 20 CLL patients an apoptosis analysis within the CD19^+^ cell population was performed. The level of apoptosis was measured by MitoTracker Red CMXRos (Thermo Fisher Scientific). CMXRos was used in combination with an anti-CD19 FITC monoclonal antibodies (BD Pharmingen, USA). Mononuclear cells were incubated with CMXRos for 30 min at 37 °C and, after 15 min of incubation, the anti-CD19 monoclonal antibody was added. The CD19^+^ cells that were defined to be apoptotic showed a decrease in the mitochondrial membrane potential following CMXRos staining (ΔΨm^low^).

### Statistical Analysis

Statistical analysis of the results was performed using Statistica 12.0 PL and GraphPad Prism 5 software. A nonparametric Mann–Whitney U test was used to compare and assess differences between the patients and control group as well as between the groups of patients in the early and advanced stages of CLL. The existence of statistical relationships between variables was evaluated by calculating Spearman’s rank correlation coefficient. Receiver operating characteristic (ROC) curves were analyzed to obtain the TLR2 expression cut-off values that best distinguished ZAP-70-positive and ZAP-70-negative cases. ZAP-70 was used in ROC curve analysis because in our previous studies ZAP-70 has been shown as one of the most powerful prognostic factors (Hus et al. 2006). Time to treatment (TTT) and overall survival (OS) distributions were plotted using Kaplan–Meier estimates. The log-rank test was used to compare the distribution. Descriptive statistics for quantitative variables included median and range. Results were considered statistically significant when the *p* value was ≤0.05.

## Results

### Membrane TLR2 Expression on CD19^+^/CD5^+^/TLR2^+^ Cells from CLL Patients and Healthy Volunteers

In our first assessment, for each sample, membrane TLR2 expression was performed on the CD19^+^ cells. Next, additional analysis for identification of CD5^+^CD19^+^ and CD19^+^CD5^−^ cells with TLR2 expression was performed (Fig. 1). No significant differences were observed in the percentage of CD19^+^/CD5^+^/TLR2^+^ (median: CLL 0.38%; healthy volunteers (HV) 1.58%) (within CD19^+^/CD5^+^ cells) and CD19^+^/CD5^−^/TLR2^+^ (within CD19^+^/CD5^−^ cells) (median: CLL 0.41%; HV 1.94%) (*p* > 0.05). Additionally, the Spearman correlation test showed a direct correlation between the results obtained using both types of analysis (*r* = 0.893). The percentage of CD19^+^/CD5^+^/TLR2^+^ cells was used in the further part of the work.

**Fig. 2:**
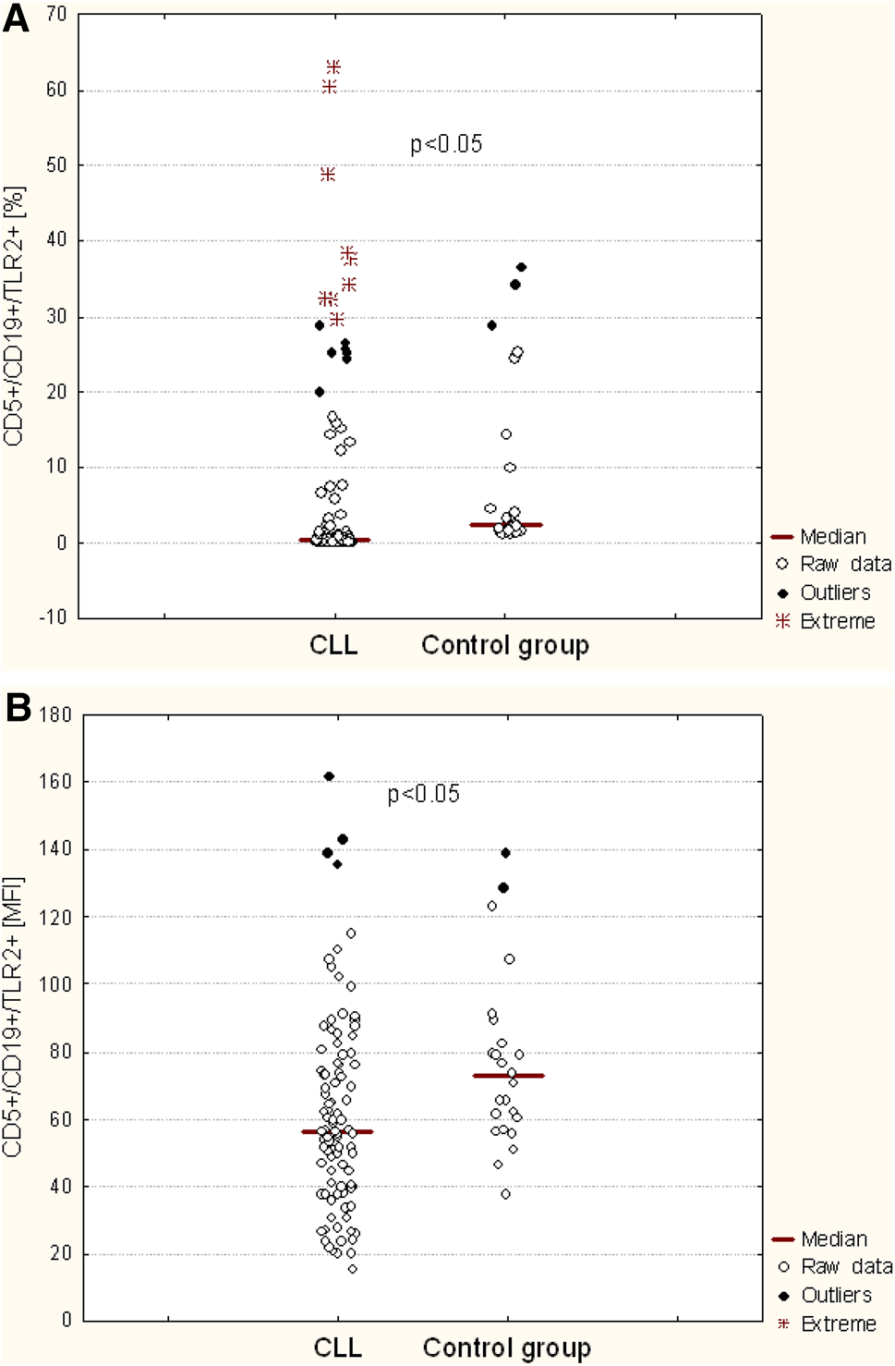
TLR2 expression on CD19^+^/CD5^+^ cells of CLL patients and control group: **a** percentage of TLR2-positive CD19^+^/CD5^+^ cells; **b** mean fluorescence intensity (MFI) of TLR2

The percentage of CD19^+^/CD5^+^/TLR2^+^ cells was significantly lower in patients with CLL as compared to the control group (*p* < 0.05) (Fig. 2a; Table 2). Likewise, when we compared the level of membrane TLR2 expression determined by MFI on B CD5^+^ cells from CLL patients and healthy volunteers, we found significant difference between the groups (*p* < 0.05) (Fig. 2b; Table 2). The percentage of CD19^+^/CD5^+^/TLR2^+^ in CLL patients was diverse and significantly higher (*p* < 0.05) in patients at stage 0 (median: 0.52%) as compared to the stages I–II (median 0.29%) and III-IV (median 0.30%) according to Rai stages (Table 2). We also observed a tendency to higher MFI in patients at stage 0 as compared to the stages I–II and III–IV, however, the difference was not statistically significant (Table 2).

**Table 2:** TLR2 expression on CD19^+^/CD5^+^ of CLL patients and healthy controls

	CD19^+^/CD5^+^/TLR2^+^ (%) Median (range)	CD19^+^/CD5^+^/TLR2^+^ (MFI) Median (range)
Healthy volunteers PB	1.58 (1.16–36.73)	72.53 (37.84–139.30)
CLL patients PB	0.38 (0.02–63.06)	39.70 (15.77–161.80)
Low risk (stage 0)	0.52 (0.04–63.06)	68.87 (20.27–161.80)
Intermediate-risk (stage I/II)	0.29 (0.03–37.76)	57.02 (20.75–143.50)
High-risk (stage III/IV)	0.30 (0.02–60.46)	37.86 (15.77–139.30)
ZAP-70^−^	1.64 (0.02–63.06)	65.52 (28.19–161.80)
ZAP-70^+^	0.26 (0.03–48.88)	45.89 (15.77–143.50)
CD38^−^	0.98 (0.02–63.06)	65.50 (28.19–161.80)
CD38^+^	0.26 (0.03–48.88)	44.28 (15.77–143.50)
del(17p13.1) and/or del(11q22.3)	0.24 (0.02–48.88)	55.88 (15.77–143.50)
Without del(17p13.1) and del(11q22.3)	0.49 (0.09–60.46)	68.84 (44.59–108.00)
Isolated del(13q14)	0.72 (0.04–63.06)	67.80 (28.19–161.80)
CLL patients requiring therapy	0.29 (0.02–60.46)	62.03 (15.77–139.25)
Untreated CLL patients	0.44 (0.04–63.06)	66.84 (31.00–161.80)

CLL patients were divided according to adverse prognostic factors, *MFI* mean fluorescence intensity, *PB* peripheral blood

### The Relationship between TLR2 and CD38 and ZAP-70 Expression

**Fig. 3:**
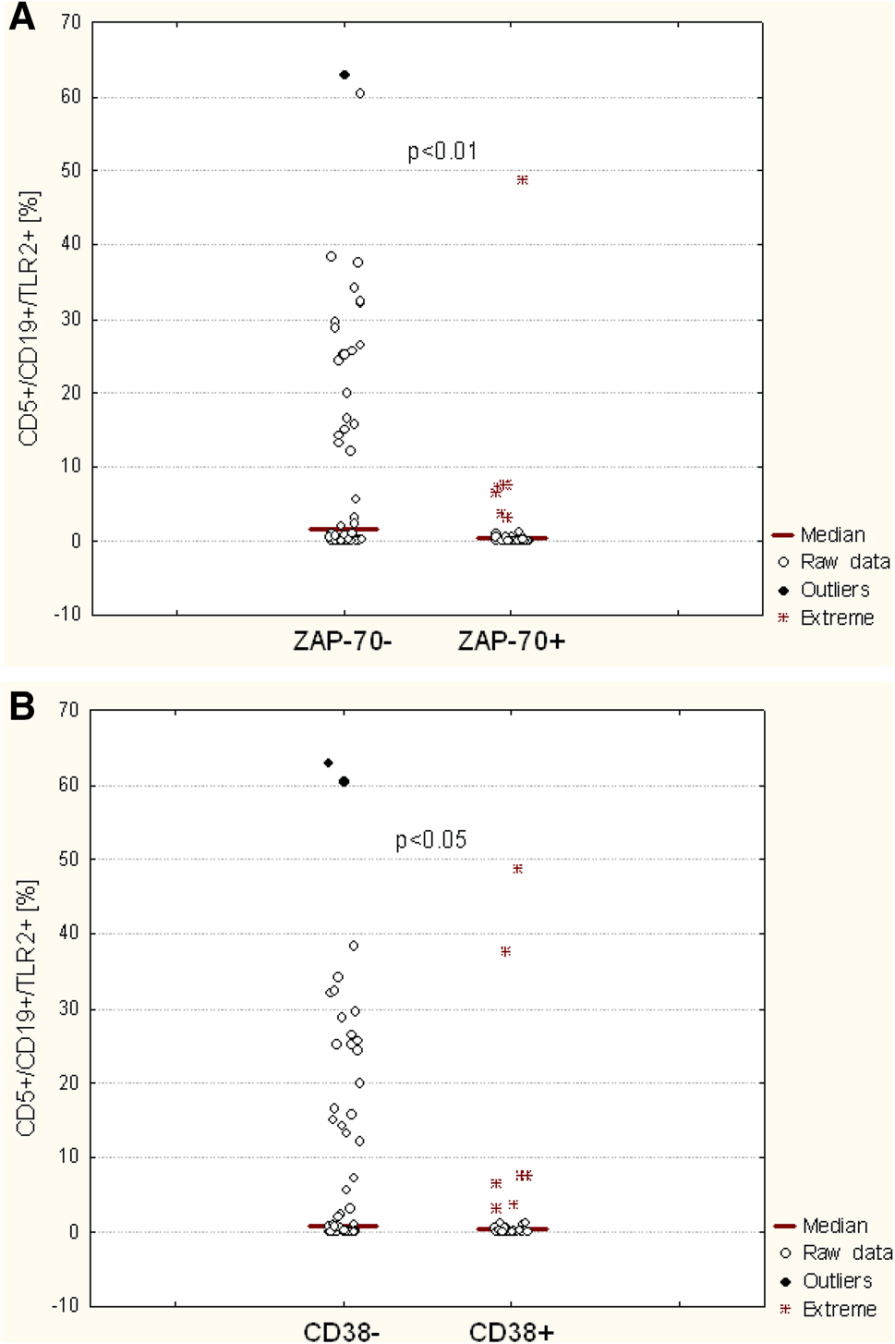
The percentage of CD19^+^/CD5^+^/TLR2^+^ in CLL patients depending on ZAP-70 (**a**) and CD38 expression (**b**)

**Fig. 4:**
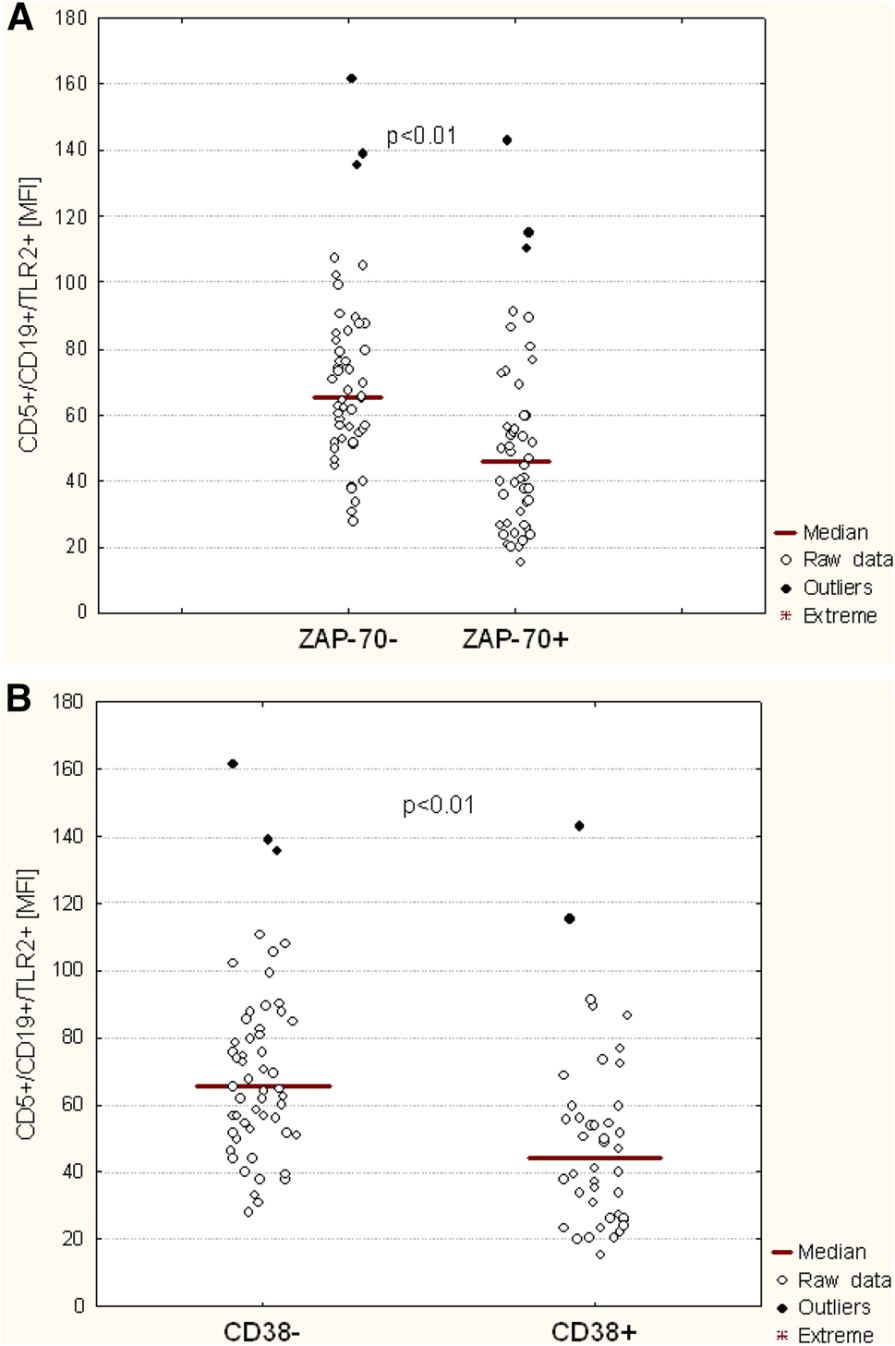
Membrane TLR2 expression determined by MFI (mean fluorescence intensity*)* in CLL patients depending on ZAP-70 (**a**) and CD38 expression (**b**)

Significant differences in the percentages of CD19^+^/CD5^+^/TLR2^+^ cells were noted in patients with CLL depending on the presence of poor prognostic factors. There was a significantly lower percentage of CD19^+^/CD5^+^/TLR2^+^ cells in ZAP-70^+^ patients compared to ZAP-70^−^ patients (*p* < 0.01) (Fig. 3A; Table 2). Likewise, higher membrane TLR2 expression determined by MFI was observed in ZAP-70^+^ than in ZAP-70^−^ patients (*p* < 0.01) (Fig. 4a; Table 2). We also observed a significantly higher percentage of CD19^+^/CD5^+^ cells with TLR2 expression in CD38-negative patients than in CD38-positive ones (*p* < 0.01) (Fig. 3b; Table 2). Likewise, MFI was higher in CD38^−^ than in CD38^+^ patients (*p* < 0.01) (Fig. 4b; Table 2).

### Membrane TLR2 Expression on CD19^+^/CD5^+^/TLR2^+^ Cells in Patients Carrying Cytogenetic Abnormalities

**Fig. 5:**
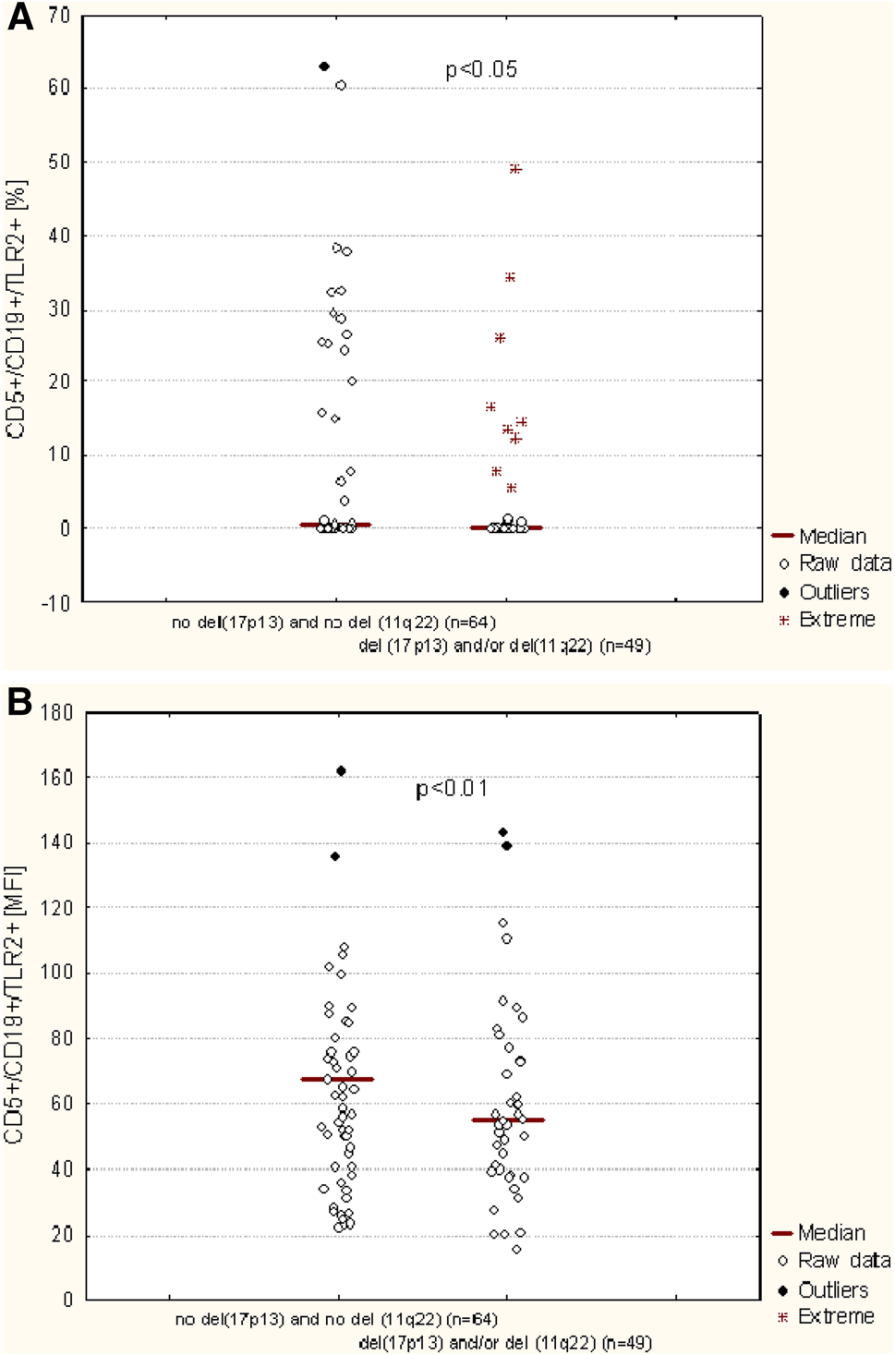
TLR2 expression on CD19^+^/CD5^+^ cells of CLL patients depending on the presence cytogenetic aberrations (del(11q22) and del(17p13)). **a** Percentage of TLR2-positive CD19^+^/CD5^+^ cells; **b** mean fluorescence intensity (MFI) of TLR2

Molecular cytogenetic analysis was available for 113 out of 119 CLL patients. The patients were divided into groups according to the results received. The first group consisted of 49 CLL patients who had del(11q22) and/or del(17p13). The second group consisted of 64 patients without these genetic changes. As shown in Table 2 and Fig. 5a, there was a significant difference in the median percentage of CD19^+^/CD5^+^/TLR2^+^ cells between patients carrying the del(11q22) and/or the del(17p13) and patients without these aberrations (*p* < 0.01). Similarly, patients carrying these unfavorable genetic changes exhibited a significantly higher membrane TLR2 expression determined by MFI comparing to the CLL patients without these abnormalities (*p* < 0.01) (Fig. 5b; Table 2).

**Fig. 6:**
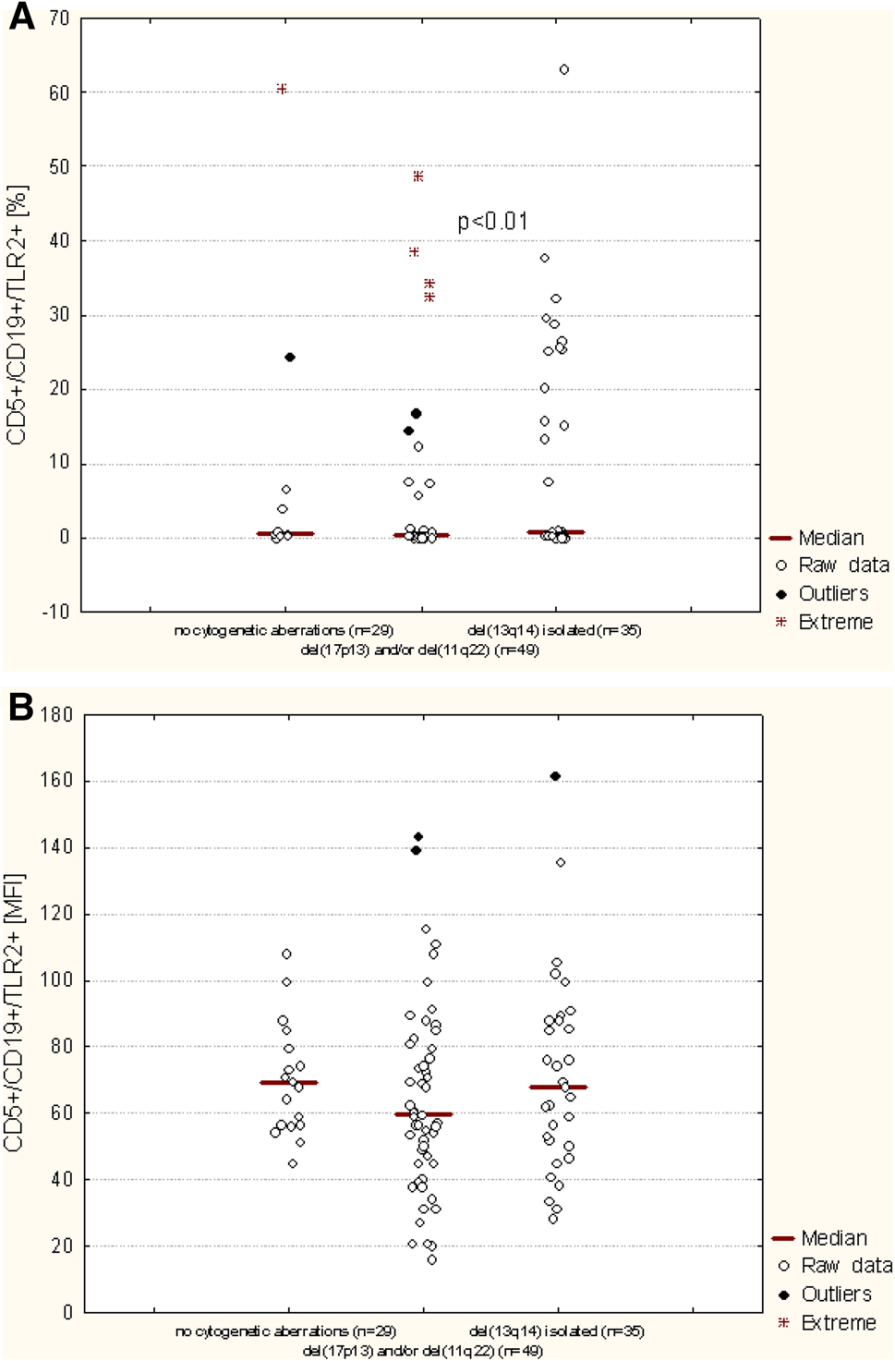
TLR2 expression on CD19^+^/CD5^+^ cells of CLL patients depending on the presence of del(13q14). **A** percentage of TLR2-positive CD19^+^/CD5^+^ cells; **b** mean fluorescence intensity (MFI) of TLR2

As the next, the percentage of B CD5^+^ cells with TLR2 expression was evaluated depending on the presence of del(13q14). The percentage of TLR2-positive CD19^+^/CD5^+^ cells was significant higher in CLL patients with isolated del(13q14) (median: 0.72%) as compared to the patients with del(11q22) and/or del(17p13) (median: 0.24%) (*p* < 0.01). There was no significant difference in the CD19^+^/CD5^+^/TLR2^+^ percentage between the patients carry isolated del(13q14) and patients without unfavorable cytogenetic aberrations (median 0.49%) (Fig. 6). Additional analysis indicated a tendency to higher membrane TLR2 expression determined by MFI in patients with isolated del(13q14) or in patients without genetic changes comparing to the patients carried del(17p13) and/or del(11q22). However, the difference was not statistically significant (Fig. 6; Table 2).

### Comparison of TLR2-Positive and TLR2-Negative Patient Groups. TLR2 Expression and Clinical Outcome of CLL Patients

Based on the ROC curve, we determined that the best threshold for the percentage of CD19^+^/CD5^+^/TLR2^+^ cells that was associated with ZAP-70 above 20% was greater than 1.6%. Using 1.6% as a cut-off value we divided our cohort into two groups: TLR2-negative (less than 1.6% of CD19^+^/CD5^+^/TLR2^+^ cells; *n* = 76) and TLR2-positive (1.6% or more of CD19^+^/CD5^+^/TLR2^+^ cells; *n* = 43) groups. TLR2-positive and TLR2-negative patients’ characteristics at the time of CLL diagnosis are summarized in Table 3. There was no significant difference between the two groups in terms of lactate dehydrogenase (LDH) levels, platelets count, β2-microglobulin and hemoglobin levels. Likewise, no significant difference (*p* > 0.05) between the TLR2^−^ and TLR2^+^ groups in lymphocyte count. However, there was significant difference between the groups in white blood cell count (*p* < 0.05). In addition, TLR2-positive patients had significantly higher percentage of leukemic cells with ZAP-70 (*p* < 0.01) or CD38 (*p* < 0.01) expression (Table 3).

**Table 3:** Clinical characteristics of TLR2-negative and TLR2-positive patients

Variable	TLR2-negative patients	TLR2-positive patients	
No. of patients (%)	76 (63.87)	43 (36.13)	
Rai stage			
Low risk (stage 0)	12 (15.79)	13 (30.23)	
Intermediate-risk (stage I/II)	48 (63.16)	25 (58.14)	
High-risk (stage III/IV)	16 (21.05)	5 (11.63)	
ZAP-70 (cut-off 20%)			
Positive (%)	37 (48.68)	14 (32.56)	
Negative (%)	39 (51.32)	29 (67.44)	
CD38 (cut-off 20%)			
Positive (%)	33 (43.42)	15 (34.88)	
Negative (%)	43 (56.58)	28 (65.12)	
Cytogenetic abnormalities			
del(17p13.1) and/or del(11q22.3)	37 (48.68)	12 (27.90)	
Isolated del(13q14)	16 (21.05)	19 (44.18)	
Without del(17p13.1) and del(11q22.3) and del(13q14)	19 (25.01)	10 (26.25)	
Not evaluated (%)	4 (5.26)	2 (4.65)	
Patients requiring therapy (%)	28 (53.4)	23 (43.49)	
Untreated patients (%)	48 (46.6)	20 (46.51)	
WBC count(G/L)^a^	32.18 (9.86–330.56)	26.65 (8.72–238.53)	*p* < 0.05
Lymphocyte count (G/L)^a^	20.26 (5.51–317.85)	18.75 (5.74–231.39)	NS
LDH (IU/L)^a^	356 (265–886)	355 (274–734)	NS
Hemoglobin (g/dL)^a^	13.5 (9.6–16.5)	13.65 (9.2–66.3)	NS
Platelets (G/L)^a^	174 (49–388)	179 (49–295)	NS
β2M (mg/dL)^a^	2.48 (1.30–8.73)	2.24 (1.45–15.20)	NS
CD19^+^/CD5^+^/ZAP-70^+^ cells (%)^a^	29.20 (0.37–64.29)	14.08 (0.21–52.38)	*p* < 0.01
CD19^+^/CD5^+^/CD38^+^ (%) cells^a^	22.40 (0.22–80.90)	11.42 (0.25–68.52)	*p* < 0.01

ROC analysis was used to determine the most significant cut-off values of TLR2 (1.6%)

*WBC* white blood cell, *LDH* lactate dehydrogenase, *β2M* β2-microglobulin, *NS* not significant

^a^Median (range)

**Fig. 7:**
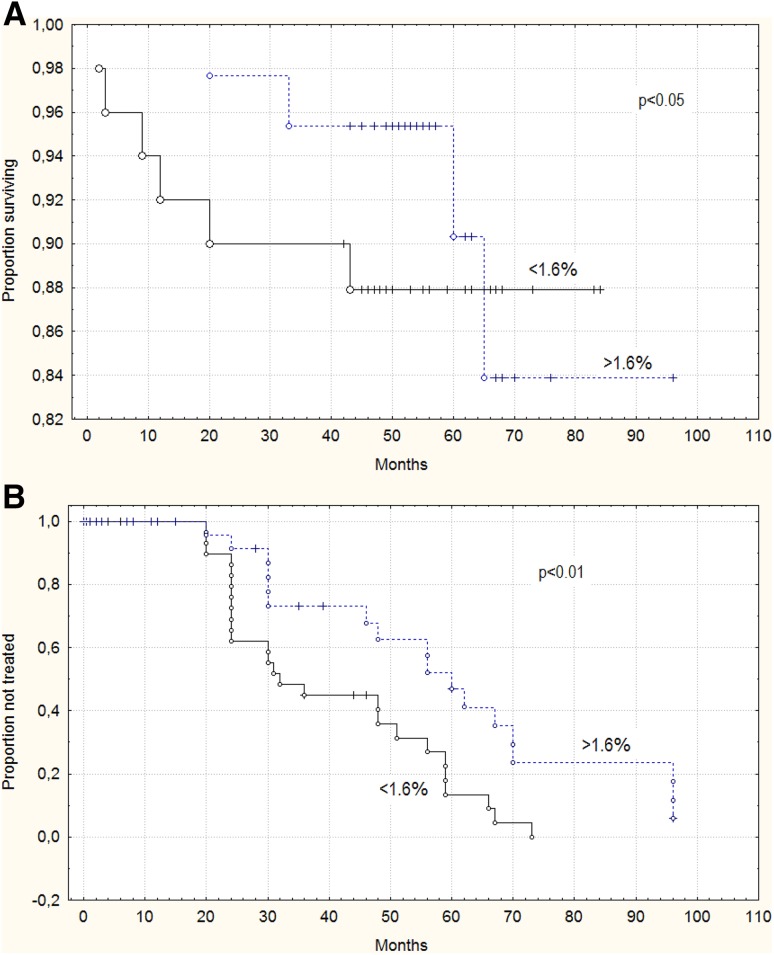
Kaplan–Meier curves based on cut-off value 1.6% for membrane TLR2 expression. **a** overall survival (OS) and **b** time to treatment (TTT) distributions in TLR2-negative and TLR2-positive groups. ROC analysis was used to determine the most significant cut-off values of TLR2 (1.6%)

Median follow-up time was 49 months (mean: 54.12 months; range: 2–96 months). During the follow-up period, the treatment was started in 51 patients (42.85%). Ten (8.4%) patients died. Time to treatment was defined as the time from date of initial diagnosis to date of first treatment. Median TTT was 24 months (mean: 22.5 months; range: 0–96 months). The median percentage of CD19^+^/CD5^+^/TLR2^+^ cells measured at the time of diagnosis was lower in patients requiring therapy (0.29%) as compared to patients without treatment (0.44%) during the observation period (*p* < 0.05) (Table 2). We also observed a tendency to lower membrane TLR2 expression determined by MFI in patients requiring therapy (62.03 MFI) comparing to the patients who did not (66.84 MFI). However, this difference was not statistically significant (*p* > 0.05) (Table 2). Figure 7a exhibits curves of OS of CLL patients depending on the cut-off (1.6%) determined based on the ROC curve. Patients with less than 1.6% of CD19^+^/CD5^+^/TLR2^+^ cells showed a shorter survival times (median: 49 months) compared to the patients with more than 1.6% of CD19^+^/CD5^+^/TLR2^+^ cells (median: 60 months) (*p* < 0.05). What is more, the group of TLR2-negative patients had a shorter TTT (median: 23 months) than TLR2-positive patients (median: 30 months) (*p* < 0.01) (Fig. 7b).

### Apoptosis

There was no significant correlation between the percentage of CD19^+^/CD5^+^/TLR2^+^ and the percentage of Δ*Ψ*m^low^/CD19^+^ lymphocytes (*r* = 206; *p* > 0.05). Likewise, no correlation was identified between the membrane expression of TLR2 determined by MFI and the percentage of apoptotic B lymphocytes determined at the time of diagnosis (*r* = 0.203; *p* > 0.05).

## Discussion

Immune system disorders are considered to play a role in the pathogenesis of CLL. Recent studies suggest that bacterial and viral infections might contribute to the induction of malignant transformation in CLL. B cells play an important role in the defense against microorganisms and induction of humoral immunity against isolated polysaccharide antigens is dependent on the presence of TLRs expressed on the innate immune cells (Isaza-Correa et al. 2014). In this study, TLR2 expression was analyzed on CD19^+^/CD5^+^ cells from peripheral blood of patients with CLL. TLR2 is the receptor responsible for recognizing the bacterial cell wall components such as lipoproteins, peptidoglycan, lipoteichoic acid. Recent studies suggest that disorders of TLRs’ expression may be important in the development of CLL (Ntoufa et al. 2016; Wagner et al. 2016), though the mechanism underlying the development of the disease still remains unexplained. We found significantly lower percentage of CD19^+^/CD5^+^TLR2^+^ cells in patients with CLL comparing to the control group that is in line with the data obtained by Grandjenette et al. (2007) and Muzio et al. (2008) who demonstrated lower expression of TLRs on leukemia cells as compared to normal B cells. What is more, in our study the MFI data indicated that leukemic lymphocytes positive for TLR2 also express less TLR2 per cell than the control CD19^+^/CD5^+^ cells. Antosz et al. (2011) observed two times lower expression of TLR2 mRNA in leukemic cells comparing to the healthy subjects. The authors suggest that the reason for immune deficiency in the clinical course of CLL may be decreased expression of TLR2 that is too low to activate co-stimulatory factors. Different results were obtained by Rybka et al. (2016), whose analysis showed higher TLR2 gene expression in leukemic cells of patients with CLL as compared to the control group and similar conclusions have been reported by Chiron et al. (2008). The authors observed a decreased induction of changes in co-stimulatory molecule expression, which could be explained by disruptions in the interaction between B and T cells. TLR2 plays an essential role in the activation of innate immunity. Increased TLR2 expression has been observed in various cancers, suggesting that TLR2 may play important roles in tumorigenesis and tumor progression. It was found that activating TLR2 promotes tumor metastasis. Although the association between the expression of TLR2 and the pathogenesis of leukemia has not yet been established (Li et al. 2014). Ntoufa at al. (2016) demonstrate that CLL cells are anergic through the BCR, and that stimulation through the TLR1/2 may break B-cell anergy. The data available on TLR2 expression in CLL are still limited (Muzio et al. 2008; Ntoufa at al. 2016; Rybka et al. 2016). Comparing literature data on TLR2 expression, significant discrepancies between the results can be observed. These differences may result from the analyses of the receptor expression at mRNA and protein level and may be also related to the different status of leukemic cell activity or even to the different cell viability levels in the individual samples. Furthermore, most studies, have examined small groups of patients, thus prevent draw unequivocal conclusions with regard to the TLR expression in CLL and evaluation possible correlations with various clinico-biological parameters. Additionally, these discrepancies may be interpreted as an evidence of increased TLR expression on CLL cells by post-transcriptional mechanisms (Arvaniti et al. 2011). The studies of Antosz et al. (2012) demonstrated that CLL cells stimulation using LPS and SAC (*Staphylococcus aureus* Cowan I) resulted in an increase in TLR2 expression. Cellular stress proteins and products of cell degradation resulting from apoptosis are classified as endogenous TLR2 ligands. Thus, it can be argued that products derived from apoptotic leukemia cells may have TLR2 inducing properties and may, therefore, stimulate the production of pro-inflammatory cytokines (Akira 2003; Albiger et al. 2007; Antosz et al. 2011). In our study the percentage of apoptotic cells (Δ*Ψ*m^low^/CD19^+^) was measured at the time of diagnosis. However, no correlation was identified between the expression of TLR2 on leukemic B cells and the percentage of apoptotic B lymphocytes. This observation, however, requires confirmation in a larger group of CLL patients.

The frequency of CD19^+^/CD5^+^/TLR2^+^ cells and staining intensity of TLR2 decreased with the stage disease. In contrast, another research group detected higher TLR2 gene expression in patients with Rai stages III and IV than in patients with early stage disease (Rybka et al. 2016). It is interesting that significantly lower expression of TLR2 was noted in CLL patients with poor prognostic factors, such as: the expression of tyrosine kinase ZAP-70 in leukemic cells, the expression of CD38 antigen on their surface and the presence of del(17p) and/or del(11q). The presence of specific chromosome abnormalities, like del(17p) and del(11q) is an unfavorable prognostic factor in patients with CLL, correlating with rapid disease progression and shortened survival time of the patients (Döhner et al. 2000; Robak 2003). Clinically, the most unfavorable disorder is deletion in the region of chromosome 17 containing TP53 suppressor gene responsible for regulating the cell cycle (Döhner et al. 2000). What is more, we were able to demonstrate a significant association of TLR2 MFI or the percentage of CD19^+^/CD5^+^/TLR2^+^ cells with del(13q14). 13q deletion occurring as the sole aberration identifies a subset of CLL patients with good prognosis (Foà et al. 2013). In our study significantly higher TLR2 expression was found in patients with del(13q14) comparing to the patients with del(11q22) and/or del(17p13) and patients without cytogenetic aberrations.

In our study membrane TLR2 expression identified at the time of diagnosis was lower in patients requiring therapy as compared to patients without treatment during the observation period. We found an association between low percentage of CD19^+^/CD5^+^/TLR2^+^ cells and shorter time to treatment. We also demonstrated the relationship between low percentage of CD19^+^/CD5^+^ TLR2-positive and OS of CLL patients. CLL patients with a proportion of 1.6% TLR2-positive B CD5^+^ cells (according to the ROC analysis) or more had a longer time to treatment and longer OS than the group with a lower percentage of TLR2 positive cells. It seems that decreased TLR2 expression occurs with disease progression. The close relationship with unfavorable prognostic markers (i.e., ZAP-70, CD38, 11q, and 17p deletion), observed in our study, suggests a potential role of TLR2 expression as a prognostic factor, though the results of our studies do not allow for a clear definition of the role of TLR2 in the development and progression of CLL.

## Conclusions

To sum up, the results of the study suggest that low TLR2 expression is associated with poor prognosis in CLL patients. The monitoring of CD19^+^/CD5^+^/TLR2^+^ cells number may provide useful information on disease activity. Level of TLR2 expression on leukemic B cells may be an important factor of immunological dysfunction for patients with CLL. Our study suggests that TLR2 could becomes potential biological markers for the clinical outcome in patients with CLL.
